# Exposure to cell phone induce oxidative stress in mice preantral follicles during in vitro cultivation: An experimental study

**DOI:** 10.18502/ijrm.v17i9.5099

**Published:** 2019-09-22

**Authors:** Najmeh Vafere Koohestani, Saeed Zavareh, Taghi Lashkarbolouki, Fariba Azimipour

**Affiliations:** ^1^School of Biology Damghan University Damghan Iran.; ^2^Institute of Biological Sciences Damghan University Damghan Iran.

**Keywords:** Ovarian follicle, Cell phone, Oxidative stress, Mice

## Abstract

**Background:**

Radiations emitting from mobile phones have been proposed to affect people’s health, mediated by various mechanisms like induction of oxidative stress.

**Objective:**

This study aims to investigate the effect of cell phone exposure on the oxidative status of mice preantral follicles (PFs) during in vitro culture.

**Materials and Methods:**

PFs (n░=░2580) were isolated mechanically from 16 to 18 day-old NMRI mice (n░=░50) and divided into control and cell phone-exposed groups. PFs were cultured for 12 days and ovulation was induced using human chorion gonadotropin. The developmental parameters including size, survival, antral cavity formation, ovulation and oocyte maturation were assessed. In parallel, enzymatic antioxidants activities, total antioxidant capacity (TAC), and Malondialdehyde (MDA) levels were evaluated.

**Results:**

The diameters and the rates of survival, antrum formation, ovulation, and metaphase II oocytes of exposed PFs to cell phone were significantly lower than those of the control group (p░≤░0.001). The PFs exposed to cell phone had significantly lower superoxide dismutase (SOD), glutathione peroxidase (GPX), and catalase (CAT) activity compared with the control group. In the cell phone exposed PFs, the TAC level was significantly lower (p░≤░0.001) and MDA levels was significantly higher (p░≤░0.001), compared tothe those of control group.

**Conclusion:**

Exposure to cell phone compromised the developmental competence of mice PFs by increasing oxidative stress.

## 1 Introduction

***This article extracted from M.Sc. Thesis. (Najmeh Vafer Koohestani)***

The widespread usage of the cell phone has led to concerns about the potentially adverse effects of its emitted radiation on reproductive health (1). The mechanism of its effects is not entirely clear, however, in general, cell phones have two influencing mechanisms, namely, thermal and non-thermal effects. In thermal effects, high frequency increases tissue temperature and damages cell development, whereas, in the non-thermal effects, the passage of its impulses destruct cell membrane integrity (2). It was, however, demonstrated that the radiation emitting from commercial cell phones have non-thermal effects (3,4). The impact of cell phone radiation probably combines thermal and non-thermal effects. Recent studies have shown a possible role of cell phone usage in male infertility (3–6). Holding a cell phone near the reproductive organs such as the testes may lead to the impairment of testicular function particularly sperm production and thereby to male infertility.

Studies have shown that cell phone radiation induces oxidative stress (OS) in in vivo condition (7). OS is the imbalance between pro-oxidants and antioxidants to overcome pro-oxidant. Cell phone radiation seems to increase the production of reactive oxygen species (ROS) by disturbing the ROS metabolism or decreasing the total antioxidant capacity (TAC) and decreasing the enzymatic antioxidant activity (8). In this regard, it was demonstrated that cell phone radiation increases mitochondrial ROS generation in human spermatozoa that lead to altering semen quality. However, previous studies on the effect of long-term exposure to cell phone radiation on Malondialdehyde (MDA) levels and enzymatic antioxidant activities revealed contradictory results (7,8). In this regard, Balci and colleagues found that cell phone-emitted radiation did not change MDA levels and superoxide dismutase (SOD), GSH-Px, and catalase (CAT) activityin lens tissue (9). While on the other hand, Oktemand colleagues showed that cell phone exposure increased MDA levels and decreased SOD, CAT, GSH-Px activities in renal tissue (8). Also, Ozgunerand colleagues demonstrated that SOD, GSH-Px, and CAT activities decreased in retina tissue of cell phone-exposed animals (10). Although previous studies showed that cell phone usage compromised male infertility (11), the effect of emitted radiation from cell phones on the female reproductive system is still unclear.

Therefore, the present study aimed to evaluate whether the cell phone radiation can affect the oxidative status and developmental competence of mice preantral follicles (PFs) during in vitro culture.

## 2 Material and Methods

### 2.1 Reagents

All chemical reagents, unless otherwise stated, were purchased from Sigma Aldrich (UK). Culture medium was created using Milli-Q water.

### 2.2 Animals

The adult female and male (6–8 wk; 20–25░g)Naval Medical Research Institute mice (NMRI; n░=░20 and 10, respectively) were housed and bred under standard conditions: 12░hr light/dark cycle and temperature condition of 24░°C with adequate food and water. Female offspring aged 16–18- day old (n░=░50) were used for all experiments.

### 2.3 Experimental design

The ovaries of mice were hold in alpha minimum essential medium (α-MEM) supplemented with 25░mM HEPES (4-(2-hydroxyethyl)-1-piperazineethanesulfonic acid) 10% FBS (fetal bovine serum; Gibco, UK), 100 IU/ml penicillin, 75░µg/mL streptomycin and 2.2░g/L sodium bicarbonate, The PFs were mechanically isolated from the ovaries as described previously (12). The PFs with with a diameter of 130–150░μm and oocyte surrounded with 2–3 layers of intact granulosa cells with intact basement membrane and at least one layer of theca cells were selected and allocated into control and experimental groups. Experimental groups were exposed to cell phone (Sony Ericsson K800) with carrier frequency of 1,900░MHz and specific absorption rate (SAR) ranged from 0.77 to 0.88░W/kg in talking mode at 5░cm distance from the culture dish containing PFs for 60░min inside the CO_2_ incubator (Memmert, Germany). The PFs were cultured for up to 12 days to evaluate the developmental parameters. In parallel, some of the PFs were randomly selected to assess the oxidative status. All experiments were repeated at least four times.

### 2.4 In vitro culture of PFs

PFs were cultured in 25░µL drops of α-MEM supplemented with 100░m IU/mL recombinant human follicle-stimulating hormone (rhFSH), 5% FBS, 1% insulin-transferring-selenium (ITS), and 20░ng/mL recombinant epidermal growth factor (rEGF) under embryo-tested mineral oil in an incubator at 37░°C in 5% CO_2_ in air for 10 days as previously described (13). Culture medium was changed every other day for 10 days. Along with the changing environment, the growth of PFs was evaluated by calculating the average of two perpendicular diameters with an inverted microscope with the precalibrated ocular micrometer on 2^nd^ and 4th culture. On the 10th day of the cultivation, culture medium was changed with 1.5 IU/ml of human chorionic gonadotropin (hCG) to induce ovulation. After 48░hr, oocytes were considered regarding maturation stages as germinal vesicle (GV), germinal vesicle breakdown (GVBD), and metaphase II oocytes (MII), as described previously (14). The antrum formation and survival rate of cultured PFs were detected by assessing PFs morphology. Every lucent area between granulosa cells was noted as the antral cavity. Also, degenerated PFs were considered as PFs with either naked oocytes or without it and the darkness of surrounding cumulus cells.

### 2.5 Evaluation of oxidative status

#### 2.5.1 Cellular supernatant preparation

For the assessment of SOD, glutathione peroxidase (GPX), and CAT activities, as well as TAC and MDA levels, cellular supernatant was prepared from isolated PFs (n░=░15 for each replicate), which were gathered from the medium at initial time and on days 2, 4, 6, 8, 10, and 12 of culture period as previously described (15). PFs were briefly pooled in the microtube containing 1,000░μL of lysis buffer (pH░=░8). Lysis buffer composed of EDTA (20░mM), Tris-HCl (10░mM), and Triton (0.25% V/V) set in pH░=░8. Afterward, sonication (50░W for 1░min) was carried out to homogenize the PFs. The cellular mixture was centrifuged at 4░°C with 10,000░g for 20░min. The cellular supernatant was then collected for biochemical investigation.

#### 2.5.2 Measurement of TAC levels

Ferric reducing/antioxidant power (FRAP) method was performed to evaluate TAC as described previously (15,16); 2░mL of the tripyridyltriazine (Merck, Germany) as working solution and 50░μL of the cellular supernatant incubated in 37░°C for 10░min were combined. Standard solutions were made using 100░mmol/L to 1,000░mmol/L of FeSO_4_. The absorbance was detected using spectrophotometer (Unico, USA) at 593░nm for 10░min. Approximately 100–1,000░mmol/L Fe^+2^ (FeSO_4_ × 7 H_2_O) was used for the standard solution. TAC was measured as mol/L.

#### 2.5.3 Assessment of lipid peroxidation

Lipid peroxidation was evaluated using MDA level as an index of lipid peroxidation based on methods previously described (15,17). The prepared reagent mixture was composed of 8.1% sodium dodecyl sulfate, 0.8% thiobarbituric acid, 20% acetic acid, and 0.76% butylatedhydroxytoluene, which were added to the cellular supernatant and incubated at 95░°C for 60░min, then immediately cooled to room temperature. Afterward, the centrifuge was performed for 10░min at 2,000░g absorbance of the resultant organic layer, which was assessed spectrophotometrically at 532░nm. MDA levels were presented as nmol/mg protein.

#### 2.5.4 Assessment of enzymatic antioxidants

SOD activity was measured following the method of (15,18). A working solution, which contains the cellular supernatant (50░μL) supplemented with methionine (14.3░mmol), nitro blue tetrazolium (NBT, 82.5░μmol), potassium phosphate buffer (50░mmol, pH 7.8), and riboflavin (2.2░μmol), was applied. The reaction was induced using a fluorescent lamp 15░cm from the test tube for 10░min. The absorbance of the reaction tube was then read spectrophotometrically at 560░nm. Control was defined with reaction mixture without the cellular supernatant exposed to fluorescent, while blank was exposed to fluorescent. An inhibition of 50% NBT reduction was considered as one unit of SOD. GPX activity was measured according to the methods of (15,19). Furthermore, 50░μL of the supernatant was supplemented with a reaction mixture containing the reaction solution consisting of glutathione (150░μL, 2░mmol), glutathione reductase (0.15 U/mL), sodium azide (0.4░mmol/L), tert-butyl hydroperoxide (t-BHP, 0.5░mmol/L), nicotinamide adenine dinucleotide phosphate (NADPH, 0.3░mmol/L), and potassium phosphate buffer (25░μL). The conversion of NADPH to NADP was defined as a GPX activity and measured with absorption changes at 340░nm in 1░min/mg protein. The specific activity of CAT was assayed based on the disintegration of hydrogen peroxide through the previously described methods (15) by calculating absorbance change in 1░min as a time unit and presented as *μ*Mol/min/mg protein. The cellular supernatant was added to the reaction mixture, which was composed of H_2_O_2_ (30░mM) and potassium phosphate buffer (10░mM, pH 7.0). Afterward, the absorbance was read spectrophotometrically at 240░nm. Blank was phosphate buffer without the cellular supernatant. The total protein concentration in the cellular supernatant for the aforementioned biochemical parameters was measured using Lowry assay methods (20).

### 2.6 Ethical consideration

The adult female and male NMRI mice were obtained from the Pasteur Institute of Iran (Tehran, Iran). Animal experiments conform to the institutional standards that fulfill and follows the Declaration of Helsinki, as revised in Tokyo 2004, and has been approved by the Animal Care and Use Committee of Damghan University (No: 122018).

### 2.7 Statistical analysis

All data were analyzed using SPSS version 24 software package for Windows (SPSS Inc., Chicago, IL, USA) throgh independent samples *T*-test, and p░<░0.05 was considered statistically significant.

## 3 Results

### 3.1 Assessment of growth

**Figure 1:**
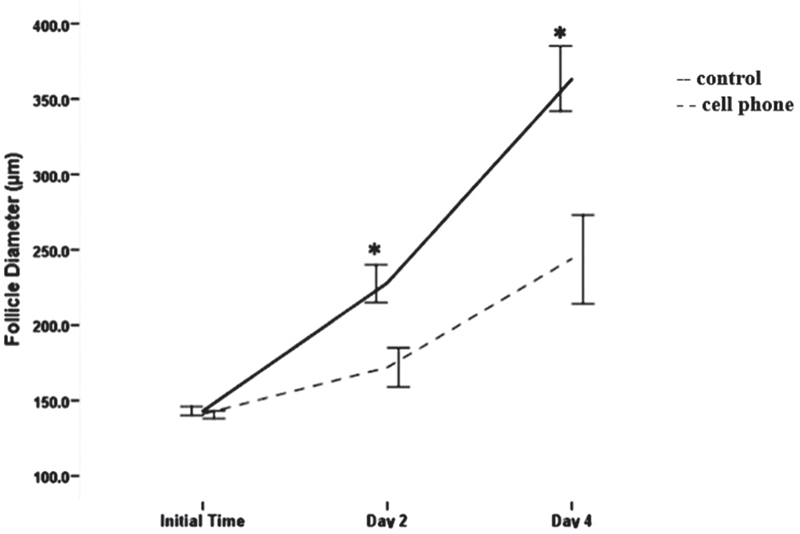
Growth changes of cultured PFs at the initial time, as well as on days 2 and 4. *Indicates significant difference.

The growth rate of PFs is shown in Figure 1. At the initial time of culture, no significant differences between the diameter of PFs in the control groups (143.33░μm) and the group exposed to the cell phone (141.08░μm, p░=░0.302) were found. On the second day, the diameter of PFs in the control group (228.00░μm) was significantly higher compared with that of the follicles exposed to the cell phone (172.35░μm, p░=░0.001). The diameter of PFs exposed to a cell phone on the fourth day (244.17░μm) was significantly lower compared with that of the control group (363.92 *μ*M: p░=░0.001).

**Figure 2:**
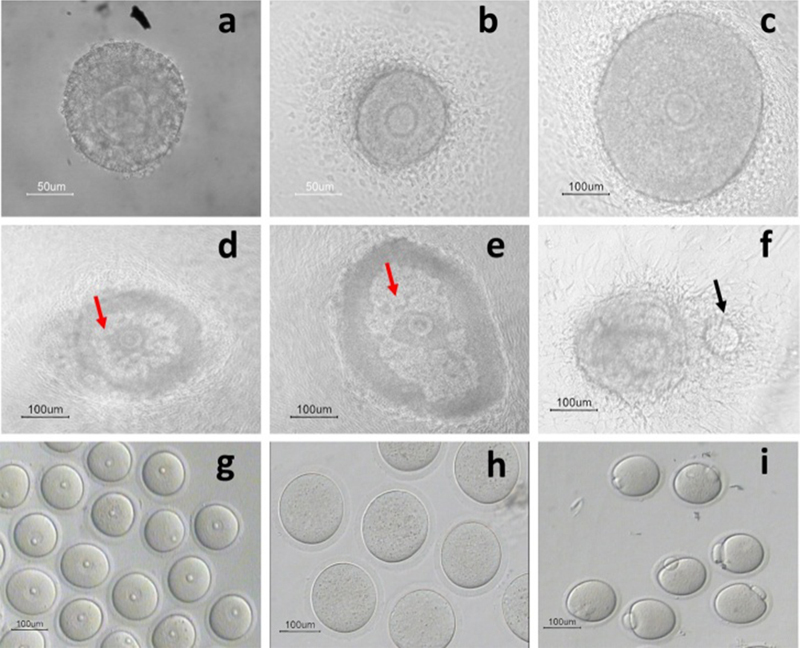
Photos of in vitro-cultured PFs on days 2 (a), 4 (b), 6 (c), 8 (d), and 10 (e), and the oocyte ovulated in cultured PFs following the addition of hCG to culture media (f) shown by the black arrow. Antrum formation is represented by the black arrow. Germinal vesicle oocytes (g), germinal vesicle breakdown in oocytes (h), and Metaphase II oocytes (i). (Preantral follicles and oocytes were visualized by inverted microscope at 400× magnification).

The developmental phases of in vitro-cultured PFs are depicted in Figure 2 and the rates of survival, antrum formation, ovulation, and oocyte maturation are summarized in Tables I and II. The rate of degenerated PFs in the control group was statistically lower compared with that of the treated group (p░=░0.003, Table I). The antrum formation rate of PFs in the control group was significantly higher than those exposed to cell phone (p░=░0.002, Table I). A significant difference (p░=░0.002,) was found between the ovulation rates of PFs in the control and cell phone-exposed groups (Table I). Furthermore, the maturation rate of harvested oocytes from control PFs was significantly higher than of those exposed to cell phones. The GVBD rate in the control group was significantly higher than that in the cell phone-exposed group (p░<░0.001, Table II). Furthermore, the rate of MII oocyte of the control group was significantly higher compared with the cell phone-exposed group (p░<░0.001, Table II).

**Table I:** The rates of developmental parameters of preantral follicles

Groups	Total	Degeneration	Antrum	Ovulation
Control	240	62 (25.83% ± 6.16)	186 (77.50% ± 6.47)	178 (74.17% ± 7.88)
Exposure to cell phone	240	^*^117 (48.75% ± 6.99)	^*^113 (47.08% ± 9.27)	^*^106 (44.17% ± 7.76)

Data presented as n (% ± SD)

* Indicates significant difference compared with the control group (p░<░0.05)

**Table II:** The rates of oocyte maturation

Groups	Total	GV	GVBD	MII
Control	240	30 (12.50% ± 2.15)	55 (22.92% ± 4.38)	93 (38.75% ± 4.38)
Exposure to cell phone	240	^*^57 (23.75% ± 6.72)	^*^16 (6.67% ± 3.36)	^*^33 (13.75% ± 2.10)

Data presented as n (% ± SD)

GV: Germinal vesicle; GVBD: Germinal vesicle breakdown; MII: Metaphase II

* Indicates significant difference compared with the controlgroup(p░<░0.05)

### 3.2 Assessment of oxidative status

**Figure 3:**
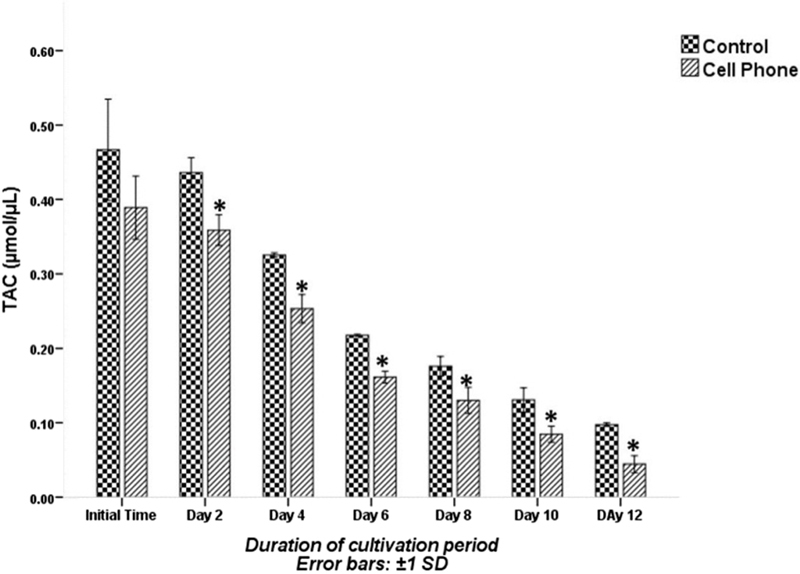
The TAC levels of PFs with or without exposure to a cell phone during the cultivation period. Data are expressed as mean░±░SD. *Indicates significant difference compared with the control group.

The TAC levels in PFs of cell phone-exposed and control groups during the cultivation period are shown in Figure 3. No significant difference was seen in TAC levels in PFs of cell phone-exposed group compared to that of the control group at the beginning of cultivation period. Whereas, on the 2nd, 4th, 6th, 8th, 10th and 12thdays of the culture period, the TAC level in the PFs of the cell phone-exposed group were significantly lower than those of the control group (p░<░0.001).

**Figure 4:**
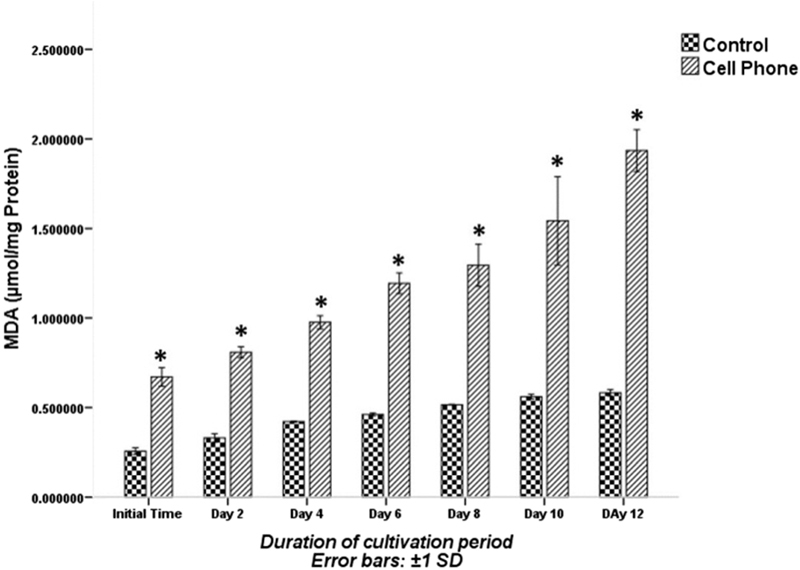
The MDA levels of PFs with or without exposure to a cell phone during the cultivation period. Data are expressed as mean░±░SD. *Indicates significant difference compared with the control group.

**Figure 5:**
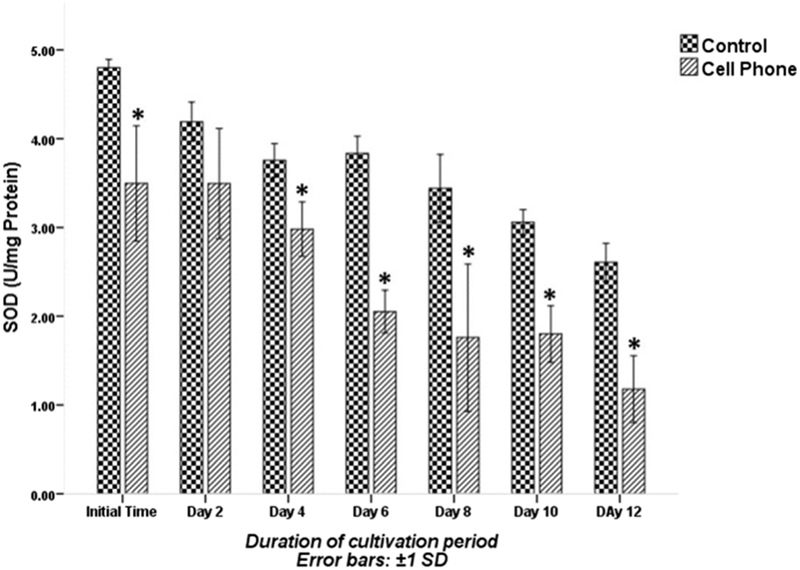
The SOD activity of PFs with or without exposure to a cell phone during the cultivation period. Data are expressed as mean░±░SD. *Indicates significant difference compared with the control group.

The MDA content in PFs of cell phone-exposed and control groups during the cultivation period is shown in Figure 4. The amounts of MDA at the initial time, the 2^nd^, 4^th^, 6^th^, 8^th^, 10^th^ and 12^th^ days of the culture period in the PFs of the cell phone-exposed group was significantly higher than those of the control group (p░<░0.001; Figure 4). The levels of SOD activity are shown in Figure 5. The SOD activity decreased in both experimental groups during the cultivation period. The SOD activity at the initial time, 4th, 6th, 8th, 10th and 12th days of the culture period in the PFs of the cell phone-exposed group was significantly lower than those of the control group (p░<░0.05), whereas the level of SOD activity on the 2nd day of the culture period was not significantly different between the control and cell phone-exposed groups (p░=░0.079).

**Figure 6:**
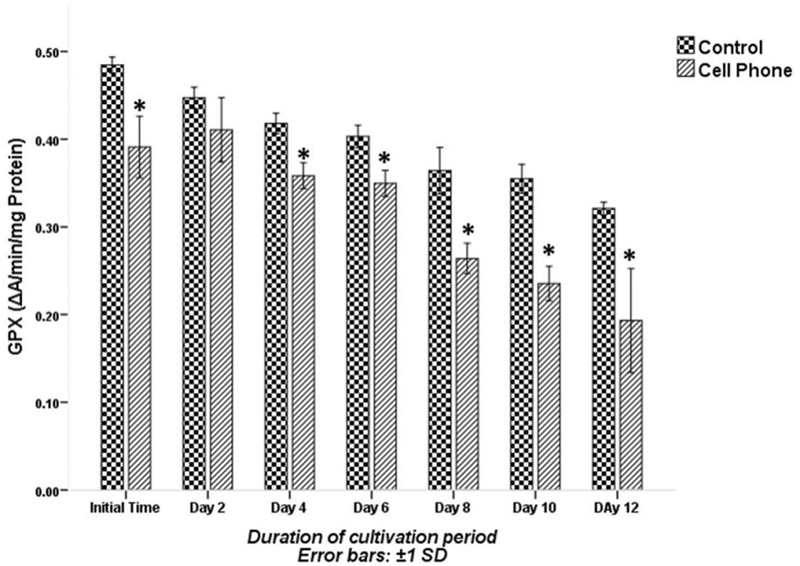
GPX activity of PFs with or without exposure to a cell phone during the cultivation period. Data are expressed as mean░±░SD. *Indicates significant difference compared with the control group.

The levels of GPX activity are shown in Figure 6. The GPX activity declined in both the experimental groups up to the end of the culture. The levels of GPX activity at the initial time and at the 4th, 6th, 8th, 10th, and 12th days of the cultivation period were significantly lower in the PFs of the cell phone-exposed group compared with the control group (p░<░0.001). Whereas, on the other hand, the level of GPX activity on the 2nd day of the culture period was not significantly different between the control and cell phone-exposed groups (p░=░0.107).

**Figure 7:**
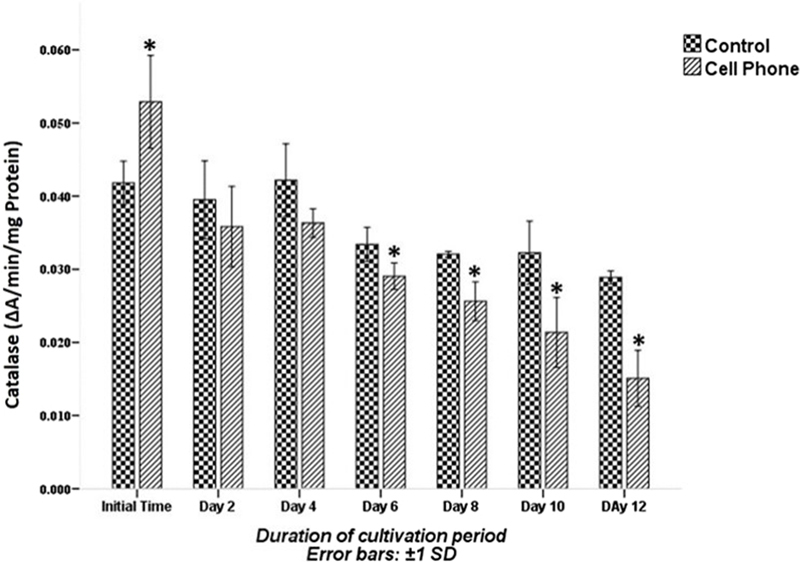
The CAT activity of PFs with or without exposure to a cell phone during the cultivation period. Data are expressed as mean░±░SD. *Indicates significant difference compared with the control group.

The CAT activity is shown in Figure 7. At the initial time of the culture period, CAT activity was significantly higher in the PFs of the cell phone-exposed group than that of the control group (p░<░0.05), whereas the CAT activity on the second and fourth days of culture in the PFs of the exposed group was not significantly different from that of the control group (p░>░0.05). The CAT activity of PFs in the cell phone-exposed group was significantly lower compared with the control group on the 6^th^, 8^th^, 10^th^ and 12^th^ days of the cultivation period (p░<░0.05).

## 4 Discussion

The results of the present study shows that the rates of the developmental parameters and enzymatic antioxidant activities of the PFs exposed to cell phone decreased significantly compared to those of the control group. In addition, the TAC and MDA levels decreased and increased, respectively, in the exposed PFs compared to those in the control group. In recent years, the use of cell phones increased the risks of exposure to electromagnetic radiation (EMR). Several studies have been conducted on the effects of electromagnetic waves on tissue damage, but conflicting results have been obtained. These contradictions can be attributed to the difference in variable frequencies, various tissues, and exposure times. The effects of EMR on fertility have several issues. The effects of EMR on male and female reproductive systems have been investigated, whereas, the mechanism of its effect is not well-known. In this regard, Safian and colleaguesshowed that the exposure to cell phone decreased the blastocysts cell viability (21), which in turn might affect normal embryonic development (22). However, EMR has been proven to cause changes to the cell cycle, enzymatic activity, and integrity of cell membrane (1,11,23). Folliculogenesis and oogenesis are the results of complex coordination between different cells, hormones, messengers, and various macromolecules. The presented data revealed that cell phone exposure has a damaging effect on the development of PFs which, in turn, diminished oocyte maturation and development. Thus, a high percentage of the ovulated oocytes from cell phone-exposed PFs were arrested at the GV stage and failed to complete nuclear maturation. Incomplete oocyte nuclear maturation, at least in part, can be explained by the EMR-induced apoptosis in somatic cells of PFs, particularly the granulosa cells and reduced proliferation (24). Although the complete mechanism of its action is unknown, another explanation could be the effect of EMR on cellular signaling, protein misfolding, and finally, cell growth inhibition (23).

Furthermore, in in vivo condition, cell phone radiation could induce OS via increased ROS production and decreased antioxidant enzyme activity (7,8). This finding is in agreement with the results of the present study, which show that the enzymatic antioxidant (SOD, GPX, and CAT) significantly altered in cultured PFs after exposure to cell phone radiation compared with those of the control group. This result is consistent with those of other investigations which showed that prolonged exposure to cell phone decreases the activities of CAT, SOD, GPX, (7). In this regard, Mao and colleagues showed that EMR disturbed gene expressions that are involved in ROS metabolism and gene-encoding antioxidant enzymes (25).

MDA level as a lipid peroxidation index is the main feature of oxidative damage. Our results show that exposure to cell phone radiation increased MDA content in PFs during the cultivation period, which, in turn, increased the production of oxidizing agents. MDA content has a reverse correlation with TAC (26), which is in agreement with our finding. Furthermore, recent studies have shown that exposure to EMR increased MDA levels and ROS production (11). In this regard, Agarwal and co-worker showed the effect of cell phone radiation on semen oxidative profiles (27). Their findings indicate that ROS production increased, followed by increased MDA and decreased TAC, SOD, GPX, and CAT in semen plasma. Moreover, in this regard, others observed that exposure to cell phones reduces enzymatic antioxidant activity (SOD and GPX) significantly, whereas a significant increase was observed in MDA levels. They concluded that excessive production of ROS was the result of cell phone exposure and had an impact on the fertility potential of sperm (11).

## 5 Conclusion

In conclusion, the present study demonstrates that the exposure to cell phone impaired the development of the mice PFs during in vitro culture through inducing OS.

## Conflicst of Interest

The authors declare that they have no conflict of interest.
